# NEIGHBORHOOD IMPACT ON OLDER ADULTS’ HEALTH OUTCOMES IN KOREA

**DOI:** 10.1093/geroni/igad104.2349

**Published:** 2023-12-21

**Authors:** Seon Kim

**Affiliations:** Virginia Commonwealth University, Richmond, Virginia, United States

## Abstract

**Background:**

The health of older adults is not only affected by biological factors but also strongly influenced by their social determinants. Neighborhood characteristics have been identified as structural contexts that significantly impact the health outcomes of older adults. As older adults tend to spend more time in their homes and neighborhoods than young adults, it is important to understand the neighborhood factors that affect their health outcomes.

**Objectives:**

This study aims to explore the neighborhood characteristics that impact the health outcomes of older adults in Korea. Method: This study used the 2020 National Survey of Older Koreans data, which was collected from 10,097 Korean older adults aged 65 and older. Neighborhood characteristics considered in this study included satisfaction with neighborhood characteristics (e.g., safety, transportation, social cohesion) and proximity to infrastructure in the neighborhood (e.g., senior centers, hospitals, supermarkets). The health outcomes considered in this study were self-rated health, the number of chronic diseases, and mental health among older adults.

**Results:**

The regression results showed that satisfaction with the neighborhood was related to older adults’ better self-rated health (B=.014, p<.001), lower number of chronic diseases (B=-.007, p<.001), and better mental health (B=.034, p<.001). On the other hand, proximity to infrastructure in the neighborhood was only related to older adults’ better mental health (B=-.018, p<.001) among older adults. Implications: These results suggest that policymakers should consider neighborhood characteristics when addressing older adults’ health outcomes. Especially, for the better mental health, policymakers should consider both proximities to infrastructures and satisfaction with the neighborhood.

